# Supplementary material for: Macro, Trace and Toxic Element Composition in Liver and Meat of Broiler Chicken Associated with Cecal Microbiome Community
Source: Biology (Basel). 2024 Nov 26;13(12):975. doi: 10.3390/biology13120975 (PMC11672959; doi:10.3390/biology13120975)
Supplement: Supplementary file 1 [file biology-13-00975-s001.zip › biology-3316872-supplementary.pdf]

Supplement S1. Description of dietary additives and modes of their use in poultry feeding

Series number	Dietary additives	Feeding regimes in control and experimental groups	References
1	*Quercus cortex* extract	Control group: basic diet (BD); experimental group I — BD + *Quercus cortex* extract 1 (1 ml/kg lw); group II — BD + *Quercus cortex* extract 2 (2 ml/kg lw); group III — BD + *Quercus cortex* extract 3 (3 ml/kg lw).	34
2	*Bacillus cereus* probiotic; coumarin	Control group: basic diet (BD); experimental group I: BD + *Bacillus cereus* probiotic (4 ml/kg lw); group II: BD + coumarin (9 mg/kg lw); group III: BD + *Bacillus cereus* probiotic + coumarin	27
3	chlortetracycline antimicrobial; *Quercus cortex* extract	Control group: basic diet (BD); experimental group I: BD + *Quercus cortex* extract (1 ml/kg lw); group II: BD + chlortetracycline (0,63 g/kg lw); group III: BD + *Quercus cortex* extract + chlortetracycline.	35
4	chlortetracycline antimicrobial; 7,8-dihydroxy-4-methylcoumarin	Control group: basic diet (BD); experimental group I: BD + chlortetracycline (0,63 g/kg lw); experimental group II: BD + 7,8-dihydroxy-4-methylcoumarin (9 mg/kg lw); experimental group III - BD + 7,8-dihydroxy-4-methylcoumarin + chlortetracycline	29
5	chlortetracycline antimicrobial; gamma lactone	Control group: basic diet (BD); experimental group I: BD + chlortetracycline (0,63 g/kg lw); experimental group II: BD + gamma lactone (0,1 ml/kg lw); experimental group III: BD + gamma lactone + chlortetracycline.	30
6	gamma lactone; vanillic acid	Control group: basic diet (BD); experimental group I: BD + gamma lactone (0.07 ml/bird/day); experimental group II: BD + vanillic acid (0.07 ml/bird/day); experimental group III: BD + gamma lactone + vanillic acid/	28
7	gamma lactone	Control group: basic diet (BD); experimental group I: BD + gamma lactone (0.05 ml/bird/day); experimental group II: BD + gamma lactone (0.2 ml/bird/day).	31
8	4-hexylresorcinol; 7,8-dihydroxy-4-methylcoumarin; gamma lactone	Control group: basic diet (BD); experimental group I: BD + 4-hexylresorcinol (0,5 mg/kg lw); experimental group II: BD + 4-hexylresorcinol (0,4 mg/kg lw) + gamma lactone (0,1 mg/kg lw); experimental group III: BD + 4-hexylresorcinol (0,1 mg/kg lw) + 7,8-dihydroxy-4-methylcoumarin (0,15 mg/kg lw); experimental group IV: BD + 4-hexylresorcinol (0,05 mg/kg lw) + gamma lactone (0,15 mg/kg lw)	26

		+ 7,8-dihydroxy-4-methylcoumarin (0,01 mg/kg lw)	
9	umbelliferon;<br>quercetin;<br>vanillin.	Control group: basic diet (BD); experimental group I: BD + quercetin (10 mg/kg daily); experimental group II: BD + quercetin + umbelliferon (2.5 mg/kg and 0.1 mg/kg per day, respectively); experimental group III: BD + vanillin (0.5 mg/kg daily); experimental group IV: BD + vanillin + umbelliferon (0.3 mg/kg each per day).	33
